# PSMC2 knockdown suppressed tumor progression of skin cutaneous melanoma

**DOI:** 10.1038/s41420-021-00727-2

**Published:** 2021-10-29

**Authors:** Yanwen Yang, Fazhi Qi, Chuanyuan Wei, Jiaqi Liu, Yong Zhang, Wenjie Luan, Jianying Gu

**Affiliations:** Department of Plastic Surgery, Zhongshan Hospital Fudan University, Shanghai, 200065 China

**Keywords:** Melanoma, Diagnostic markers

## Abstract

Skin cutaneous melanoma (SKCM) is the most lethal tumor among three of the major malignant cancers of the skin. The mechanism underlying the malignant biological behaviors of SKCM is not fully clear. Our study intended to verify the molecular mechanism of proteasome 26 S subunit ATPase 2 (PSMC2) in malignant biological behaviors of SKCM. The Cancer Genome Atlas (TCGA) database was used to analyze the expression of PSMC2 in SKCM and its impact on prognosis. PSMC2 expression in 105 paired SKCM tissues was investigated by immunohistochemistry (IHC), its functional roles were verified using a series of cell experiments, and the underlying pathway was detected by protein-chip technology and gene set enrichment analysis. We found that PSMC2 was significantly upregulated in SKCN patients from TCGA datasets and verified in clinical SKCM tissues. Moreover, high PSMC2 was shown to closely correlate with the pathological stages and lymphatic metastasis of SKCM patients. Functionally, knockdown of PSMC2 suppressed the progression of SKCM through inhibiting cell proliferation, migration, and DNA damage in vitro as well as cell growth in vivo, whereas inducing apoptosis, cycle arrest in G2 phase. Similarly, pharmaceutical inhibition of proteasome with MG132 mimicked the PSMC2 knockdown induced defects in cell cycle arrest, apoptosis and proliferation, while overexpression of PSMC2 has the opposite effects. Mechanistically, the silence of PSMC2 remarkably elevated the pro-apoptotic proteins DR6, IGFBP-4, p21, and p53, while inhibited the anti-apoptosis protein TRAILR-3 and the proteins related to the Wnt signaling pathway. The present study revealed that PSMC2 participated in a positive regulation to promote the progression of SKCM through regulating the Wnt signaling pathway. Our findings may offer a new mechanism underlying the development and progression of SKCM, and a deeper understanding of PSMC2 may contribute to SKCM treatment.

## Highlights

1. PSMC2 was found to be significantly upregulated in SKCM patients.
2. High PSMC2 expression was shown to closely correlate with the pathological stages and lymphatic metastasis of SKCM patients.
3. Knockdown of PSMC2 suppressed the progression of SKCM through inhibiting cell proliferation, migration, and DNA damage in vitro as well as cell growth in vivo, whereas inducing apoptosis, cycle arrest in the G2 phase.
4. Mechanistically, the silence of PSMC2 remarkably elevated the pro-apoptotic proteins DR6, IGFBP-4, p21, and p53, while inhibited the anti-apoptosis protein TRAILR-3 and the proteins related to the Wnt signaling pathway.

## Introduction

Cutaneous melanoma (CM) is a common skin malignant tumor with a high fatality rate, degree of malignancy, rapid progress, strong invasiveness, and poor prognosis [1]. Epidemiological studies show that the incidence of SKCM is gradually increasing with more than 20,000 new cases of SKCM every year and is one of the more rapidly improving incidence rates among malignant tumors in China [2]. Surgery is the primary and standard treatment for SKCM [3]. After surgical treatment, the 5-year survival rate of SKCM is as high as 90% in the early stage (I, II) when there are no specific clinical manifestations, but unfortunately, most SKCM patients diagnosed is an advanced stage when the 5-year survival rate is less than 10% [4, 5]. There is no standard therapy for advanced-stage SKCM. Although great progress was recorded in both the understanding of SKCM biology and genetics and biological immunization therapies of early diagnosis and prevention, this malignancy became a burden on health care worldwide due to its increasing incidence and the lack of effective advanced treatment [6]. Usually, many clinical features of patients with SKCM in China differ from SKCM patients in the West [3]. Therefore, it is essential to explore the pathogenesis and seek new therapy for patients with SKCM.

Abnormal expression of biomarker proteins can be used as the basis for early diagnosis of SKCM. Growing evidence showed that proteasome 26 S subunit ATPase 2 (PSMC2) was able to promote tumor progression as a functional gene among several types of human tumors, including ovarian cancer [7], colorectal cancer (CC) [8], osteosarcoma [9], pancreatic cancer [10], and hepatocellular carcinoma [11]. PSMC2 is a vital member of the 19 S regulatory subunit of the 26 S proteasome located on 7q22.1-q22.3 in the genome; it participated in regulating cell differentiation and proliferation, apoptosis, DNA damage repair, energy metabolism, and signal transduction [12]. PSMC2 suppression decreased cell proliferation in ovarian cancer and was also correlated with pancreatic cancer cell proliferation and apoptosis [7, 10]. PSMC2 was expressed highly in osteosarcoma patients; knockdown of PSMC2 reduced cell proliferation, migration, and increased apoptosis, which indicated that PSMC2 act as an oncogene for osteosarcoma [9]. Whether PSMC2 is involved in the occurrence and development of SKCM has not been investigated.

Here in this study, we first proved the abnormal expression of PSMC2 in SKCM tissue using gene-chip technology, then explored the biological significance and clinical relevance of PSMC2 with SKCM patients, and exam the underlying molecular mechanism of PSMC2 in SKCM last.

## Results

### A high level of PSMC2 was closely associated with clinical characteristics of patients with SKCM

**Fig. 1:**
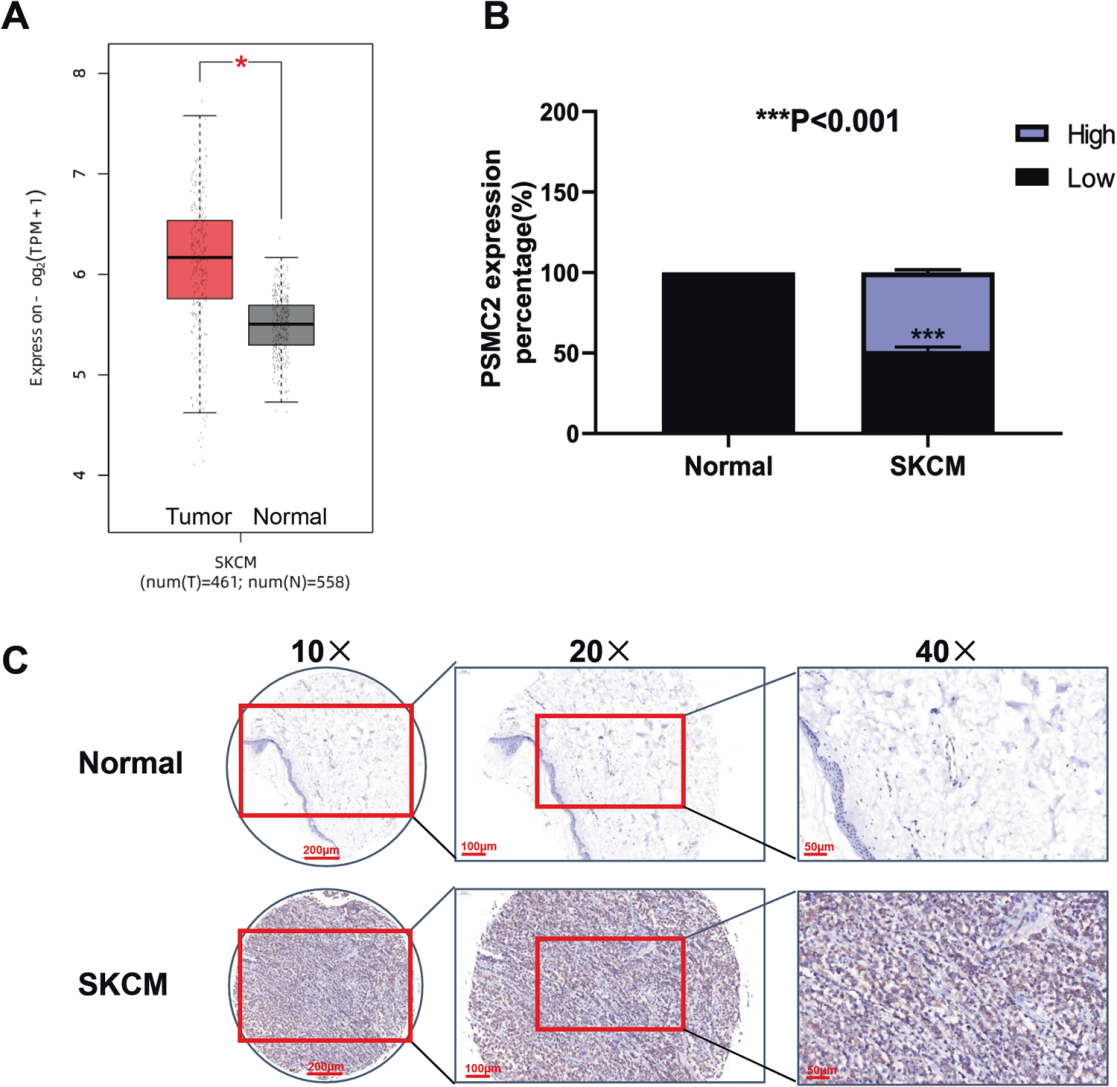
PSMC2 expression in SKCM. **A** The expression of PSMC2 was detected from the TCGA dataset by using GEPIA2 (http://gepia.cancer-pku.cn/). Red represents tumor samples and gray means normal samples. **B** Immunohistochemistry (IHC) revealed higher expression patterns of PSMC2 in SKCM tissues than that in normal skin tissues. *N* = 105. **C** Representative images of IHC staining for PSMC2 expression in SKCM and normal tissues. Data were expressed as the mean ± SD. *P < 0.05 and ***P < 0.001.

To determine the expression of PSMC2 in SKCM, we first analyzed the mRNA expression profiles and the associated clinical characteristics in 461 cases of SKCM samples compared to 558 normal samples from The Cancer Genome Atlas (TCGA) dataset. A striking upregulation of PSMC2 were observed in SKCM samples (Fig. 1A, *P* < 0.05) from TCGA, which might imply an oncogenic role of PSMC2 in the development of SKCM. Next, we determined the expression level of PSMC2 in the SKCM clinical samples, which were collected from our hospital. As shown in Fig. 1B, C, the expression of PSMC2 was significantly upregulated in human SKCM samples compared with the normal para-cancer skin tissues. Clinically, we were pleasantly surprised that SKCM patients with high PSMC2 expression were positively related to the pathological stages and lymphatic metastasis (N) (Table 1). These results suggested that PSMC2 was increased with the deepening of the malignancy degree of SKCM and might serve as a potential prognostic marker for patients with SKCM.

**Table 1:** Relationship between PSMC2 expression and clinical characteristics in patients with skin cutaneous melanoma.

Features	No. of cases	PSMC2 expression	high	*p* value
		low		
All cases	105	60	45	
Age (years)				0.104
≤ 54	54	35	19	
å 54	51	25	26	
Gender				0.611
Male	59	35	24	
Female	46	25	21	
T Infiltrate				0.074
T2	6	3	3	
T3	28	12	16	
T4	71	45	26	
lymphatic metastasis (N)				0.000***
N0	91	60	31	
N1	10	0	10	
N2	4	0	4	
Stage				0.000***
I	6	3	3	
II	83	57	26	
III	12	0	12	
IV	4	0	4	

### Knockdown of PSMC2 suppressed cell proliferation, migration, apoptosis, and DNA damage in SKCM cells, as well as cell growth in vivo

**Fig. 2:**
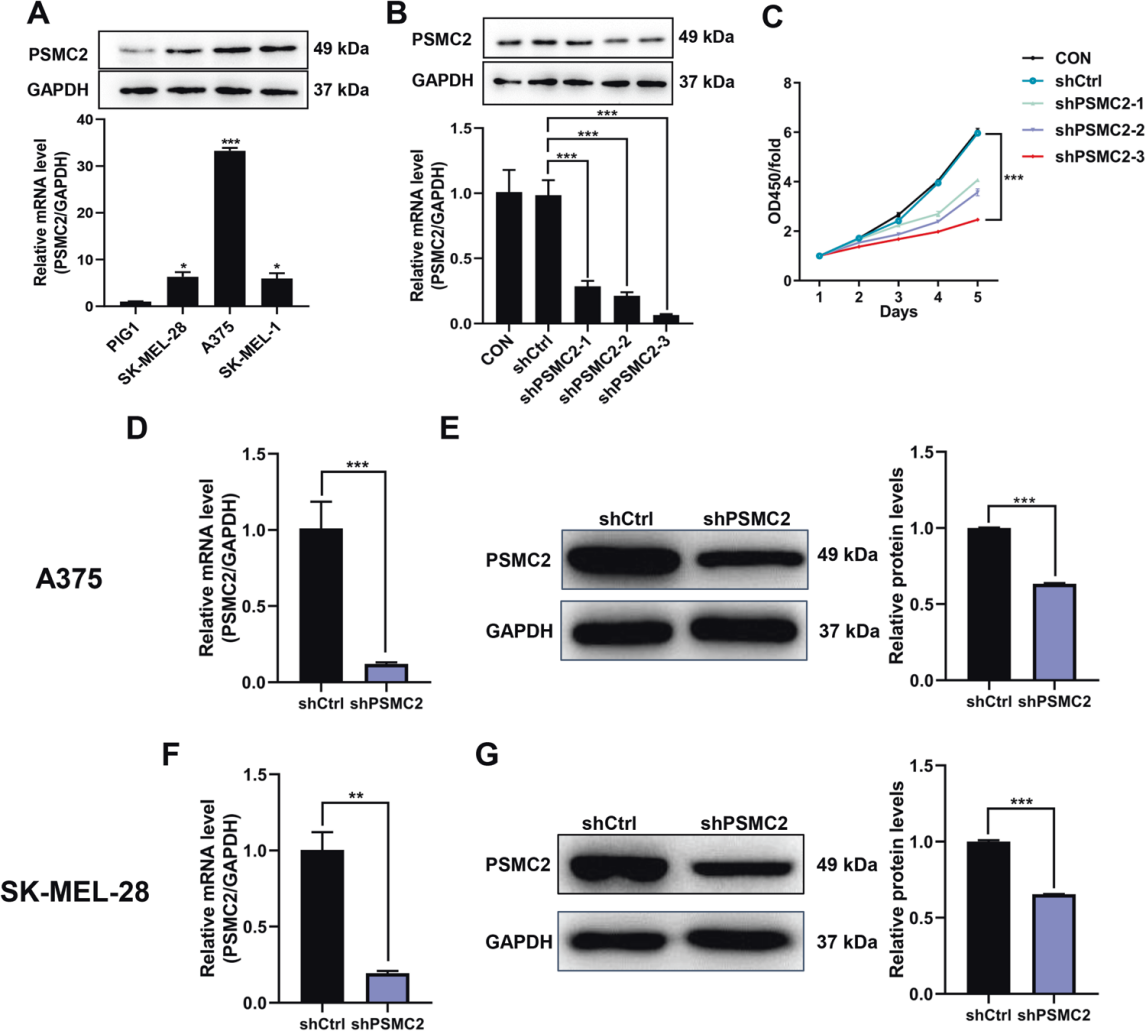
PSMC2 shRNAs transfections. **A** The PSMC2 expression profile at the protein level (upper part) and mRNA level (bottom part) in human melanoma cell lines (A375, SK-MEL-1, SK-MEL-28) and human epidermal melanocytes (PIG1). **B** Western blot (upper part) and RT-qPCR (bottom part) were used to assess the transfection efficiency of PSMC2 shRNA-1 and shRNA-2 and shRNA-3 in A375 cells. **C** PSMC2 knockdown is associated with reduced proliferation in A375. CCK-8 assay was performed to measure the rate of cell proliferation of A375 after transfected with shPSMC2-1, shPSMC2-2, and shPSMC2-3 for 5 days. **D**, **E** RT-qPCR and Western blot were used to detect the expression of PSMC2 in A375 cells after shPSMC2 transfection. **F**, **G** RT-qPCR and Western blot were used to detect the expression of PSMC2 in SK-MEL-28 cells after shPSMC2 transfection. Each independent experiment was repeated at least three times. Data were expressed as the mean ± SD. *p < 0.05, **p < 0.01 and ***p < 0.001.

**Fig. 3:**
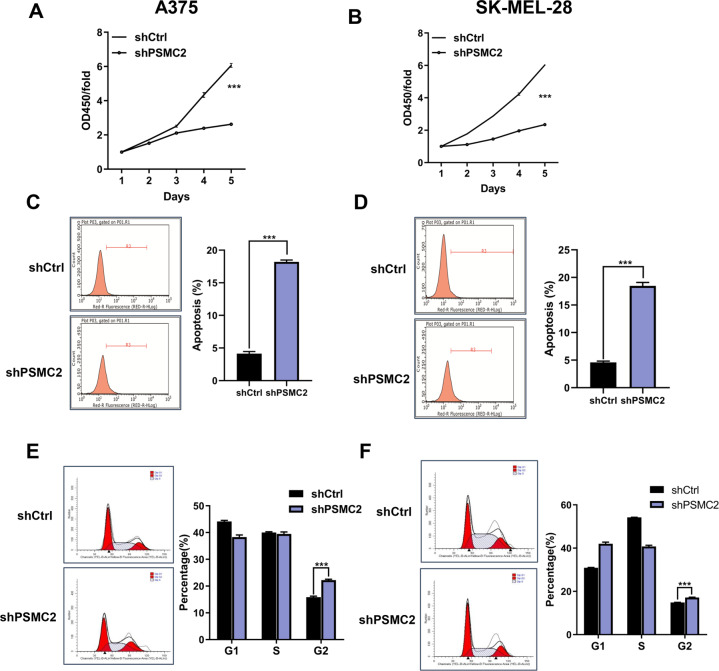
Silencing of PSMC2 suppressed cell proliferation, induced cell apoptosis and regulated cell cycle in SKCM cells. **A**, **B** CCK-8 assay was performed to measure cell proliferation of A375 and SK-MEL-28 cells after transfected with shPSMC2-1 for 5 days. **C**, **D** Apoptosis was determined by flow cytometry assays in A375 and SK-MEL-28 cells with shPSMC2 transfection and control cells. The apoptotic rate was calculated as the percentage of Annexin APC positive cells. **E**, **F** Cell cycle was determined in A375 and SK-MEL-28 cells by flow cytometry five days after treatment with shPSMC2. The diagrams quantified cell fractions in the G1, S, and G2/M fractions were shown. Each independent experiment was repeated at least three times. Data were expressed as the mean ± SD. ***p < 0.001.

**Fig. 4:**
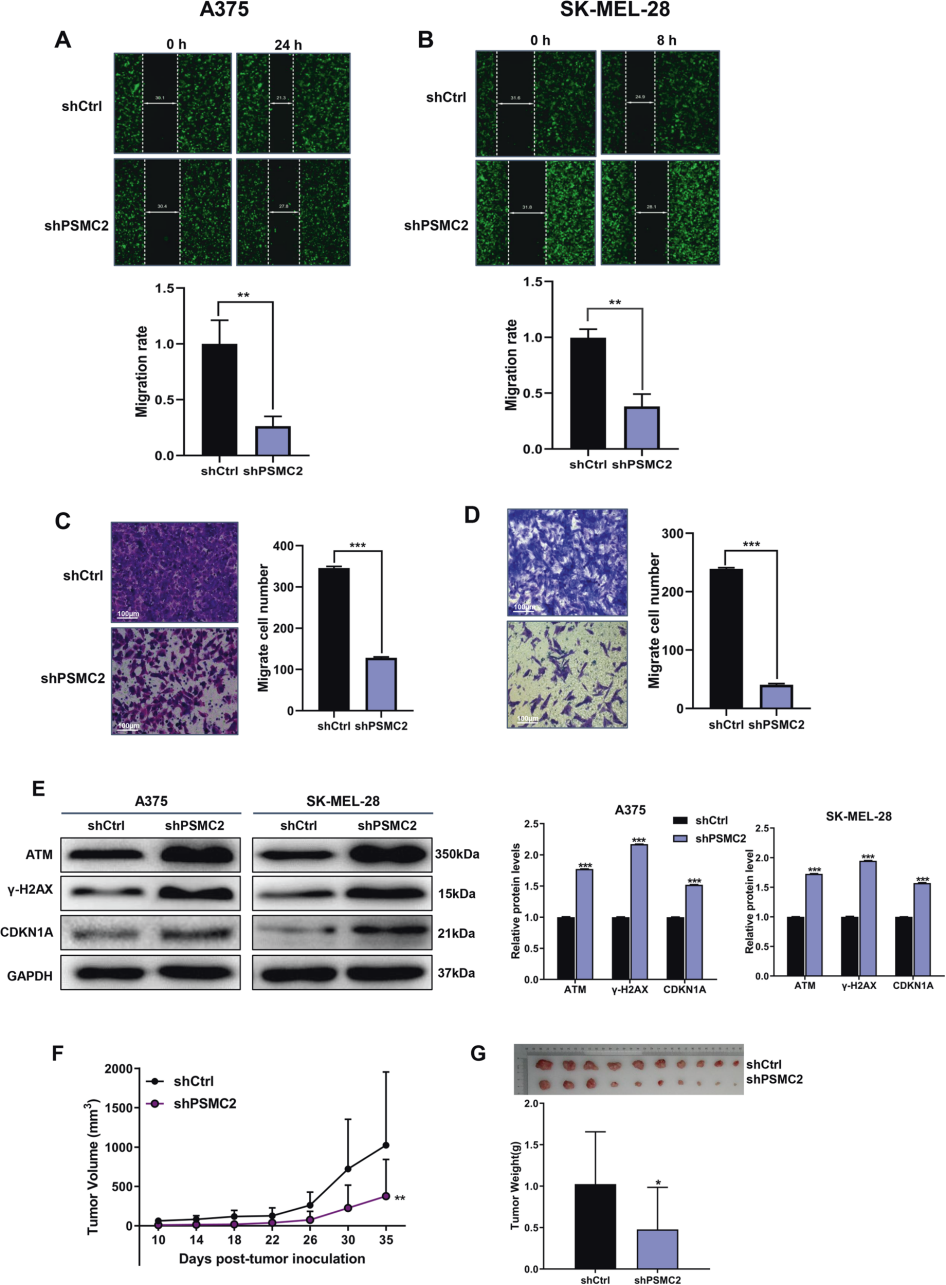
Knockdown of PSMC2 inhibits cell migration and DNA damage in SKCM cells and tumor growth in vivo. **A**, **B** Scratch test assays were performed to detect cell migrative capacities of A375 (at 0 and 24 h) and SK-MEL-28 cells (at 0 and 8 h) after shPSMC2 transfection. **C**, **D** Transwell assays were performed to detect cell migrative capacities of A375 and SK-MEL-28 cells after shPSMC2 transfection. **E** Western blotting was used to evaluate the protein expressions of ATM, γ-H2AX, and CDKN1A in PSMC2 silenced A375 and SK-MEL-28 cells. GAPDH was used as a loading control. Histogram representing indicated the results of three independent experiments. **F** Tumor volume growth curves for subcutaneous xenografts and data were obtained from 10 animals in each group. **G** After scarification, xenograft excised tumors were recorded and the bar graph presented the excised tumor weights derived from control or PSMC2 silenced A375 cells. *n* = 10. Each independent experiment was repeated at least three times. Data were expressed as the mean ± SD. The statistical significance of the data between two groups was compared using Student’s *t*-test. *p < 0.05, **p < 0.01 and ***p < 0.001.

Firstly, higher PSMC2 levels were observed in three human SKCM cell lines (A375, SK-MEL-1, and SK-MEL-28) comparing to the human normal epidermal melanocytes (PIG1) (Fig. 2A). After showing the overexpression of PSMC2 in SKCM cells, we then investigated the potential biological roles of PSMC2 in SKCM through a series of loss-of-function assays. Due to A375 cells owning relatively high PSMC2 levels, three lentivirus-mediated shRNAs targeting PSMC2 (shPSMC2–1, shPSMC2–2, and shPSMC2–3) were constructed to knockdown PSMC2 in A375 cells. After validating transfection efficiency through detecting the PSMC2 expression and cell proliferation (Fig. 2B, C), PSMC2 shRNA-3 was selected for the subsequent experiments and then collectively called shPSMC2. Subsequently, shPSMC2 effectively suppressed the expression of protein and mRNA levels both in A375 (Fig. 2D, E) and SK-MEL-28 cells (Fig. 2F, G). As predicted, knockdown of PSMC2 both in A375 and SK-MEL-28 cells was ready to suppress cell proliferation by CCK-8 assay (Fig. 3A, B). The lessened cell growth could be attributed to cell cycle distribution disorder and increased apoptosis [13]. Flow cytometry was performed to further analyze cell apoptosis and cell cycle distribution in SKCM cells to verify the above point. Furthermore, we found that PSMC2 knockdown elicited a remarkable increase of apoptotic population of A375 and SK-MEL-28 cells (Fig. 3C, D). Besides, the depletion of PSMC2 in A375 cells leads to a significant arrest in the G2 phase (Fig. 3E). Similarly, an increasing cells population in the G2 phase was also observed in SK-MEL-28 cells transfected with shPSMC2 (Fig. 3F). Next, we found that PSMC2 knockdown remarkably inhibited cell migration of A375 and SK-MEL-28 cells through wound healing and Transwell assays (Fig. 4A–D). Due to γ-H2AX, ATM and CDKN1A were the key proteins response to DNA damage, we found that PSMC2 depletion could remarkably inhibit the protein expression of γ-H2AX, ATM, and CDKN1A both in A375 and SK-MEL-28 cell lines (Fig. 4E). Meanwhile, the results of the tumor-forming experiment in nude mice showed that the tumor volume and weight in the shPSMC2 group were significantly lower than those in the control group (Figs. 4F, G). These results strongly support that the silencing of PSMC2 contributes to inhibiting malignant phenotypes and tumor progression of SKCM in vitro and in vivo.

### Inhibiting PSMC2 induces SKCM cell growth suppression, increased cell cycle arrest, and enhances apoptosis, while overexpression of PSMC2 promote SKCM growth

**Fig. 5:**
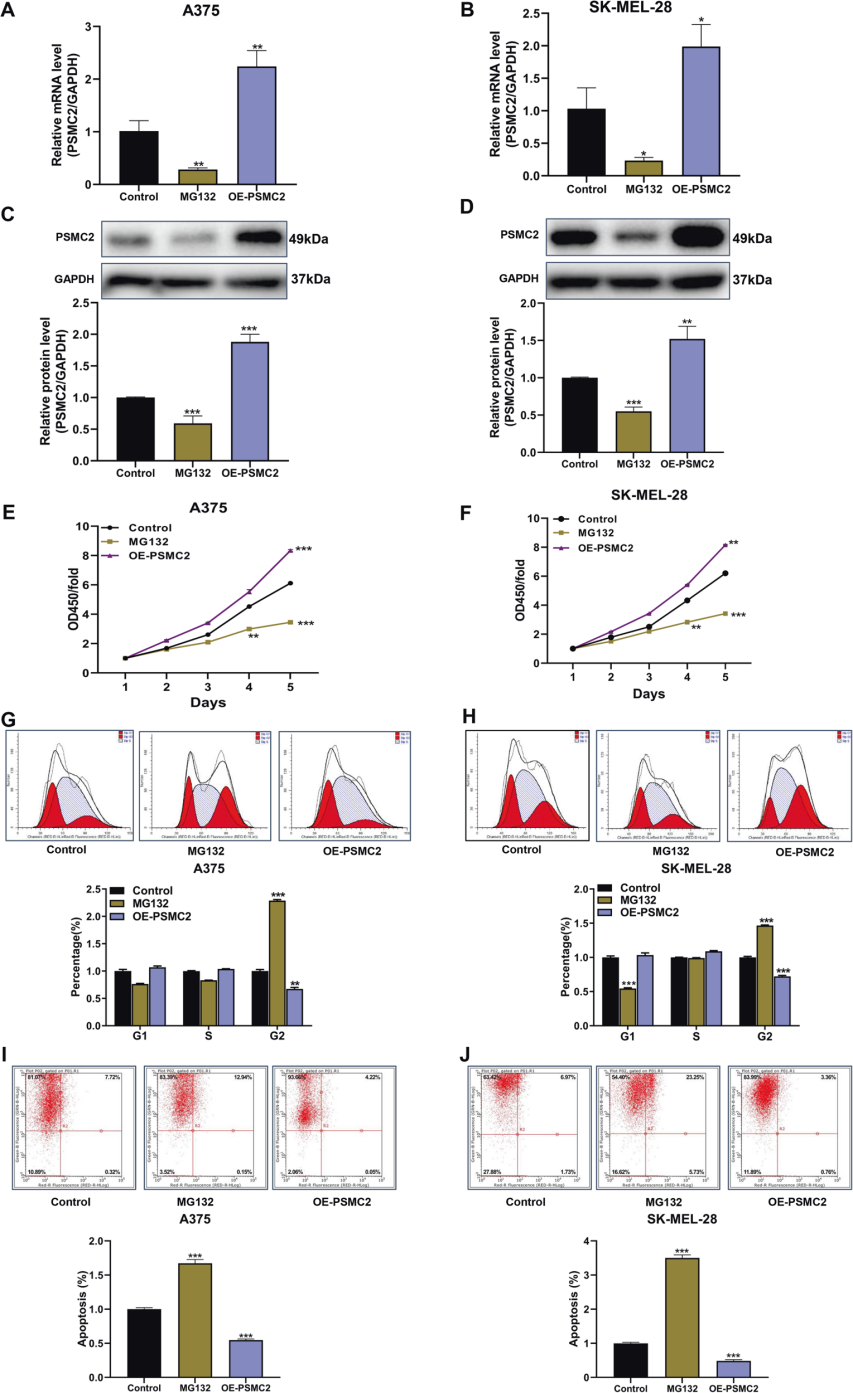
The effect of inhibiting proteasome and exogenously enforced PSMC2 expression on cell proliferation, cell cycle and apoptosis. After treatment with MG132 or OE-PSMC2 plasmids, (**A**, **B**) The mRNA levels of PSMC2 were assessed by real-time PCR in A375 and SK-MEL-28 cells. **C**, **D**) The protein levels of PSMC2 were determined by western blot in A375 and SK-MEL-28 cells. **E**, **F** The consequent influences on SKCM cell proliferation of A375 and SK-MEL-28 cells were separately determined by the CCK-8 assay. **G**, **H** Flow cytometry analysis was used to evaluate the cell cycle progression and cell fractional quantification in the G0/G1, S, and G2/M phases were shown in the diagrams. **I**, **J** Apoptosis via quantifying Annexin FITC positive cells were shown and the apoptotic rate was calculated as the percentage of Annexin APC positive cells. Each independent experiment was repeated at least three times. Data were expressed as the mean ± SD. *p < 0.05, **p < 0.01 and ***p < 0.001.

As PSMC2 is a crucial element for proteasome assembly and functional performance, we deduced PSMC2 depletion causing biological abnormalities in SKCM cells might associate with disabled proteasome activity. To explore this surmise, MG132, a selective inhibitor of the proteasome, was added to A375 and SK-MEL-28 SKCM cells to down-regulate PSMC2 expression (Fig. 5A–D). Meanwhile, lentivirus transduction (OE-PSMC2) was used to specifically upregulate PSMC2 expression in two SKCM cells (Fig. 5A–D). Expectedly, MG132 was able to suppress cell growth (Fig. 5E, F), while to elevate the cell population in the G2/M phase (Fig. 5G, H), and promoting cell apoptosis (Fig. 5I, J). In addition, overexpression of PSMC2 remarkably promoted cell growth of A375 and SK-MEL-28 cells via CCK-8 assay (Fig. 5E, F). Inconsistent, cell population in the G2/M phase was obviously suppressed in PSMC2 overexpressing cells (Fig. 5G, H), as well as cell apoptosis was restrained (Fig. 5I, J). Herein, we proved proteasome is necessary for SKCM growth due to its inhibition or deficiency for PSMC2 would lead to the biological function defects of SKCM cells. Additionally, these findings also indicated that exogenous enhanced PSMC2 expression could apply a valid effect on SKCM growth.

### Silencing of PSMC2 alters apoptosis-related gene expression and enrichment of related pathways in SKCM cells

**Fig. 6:**
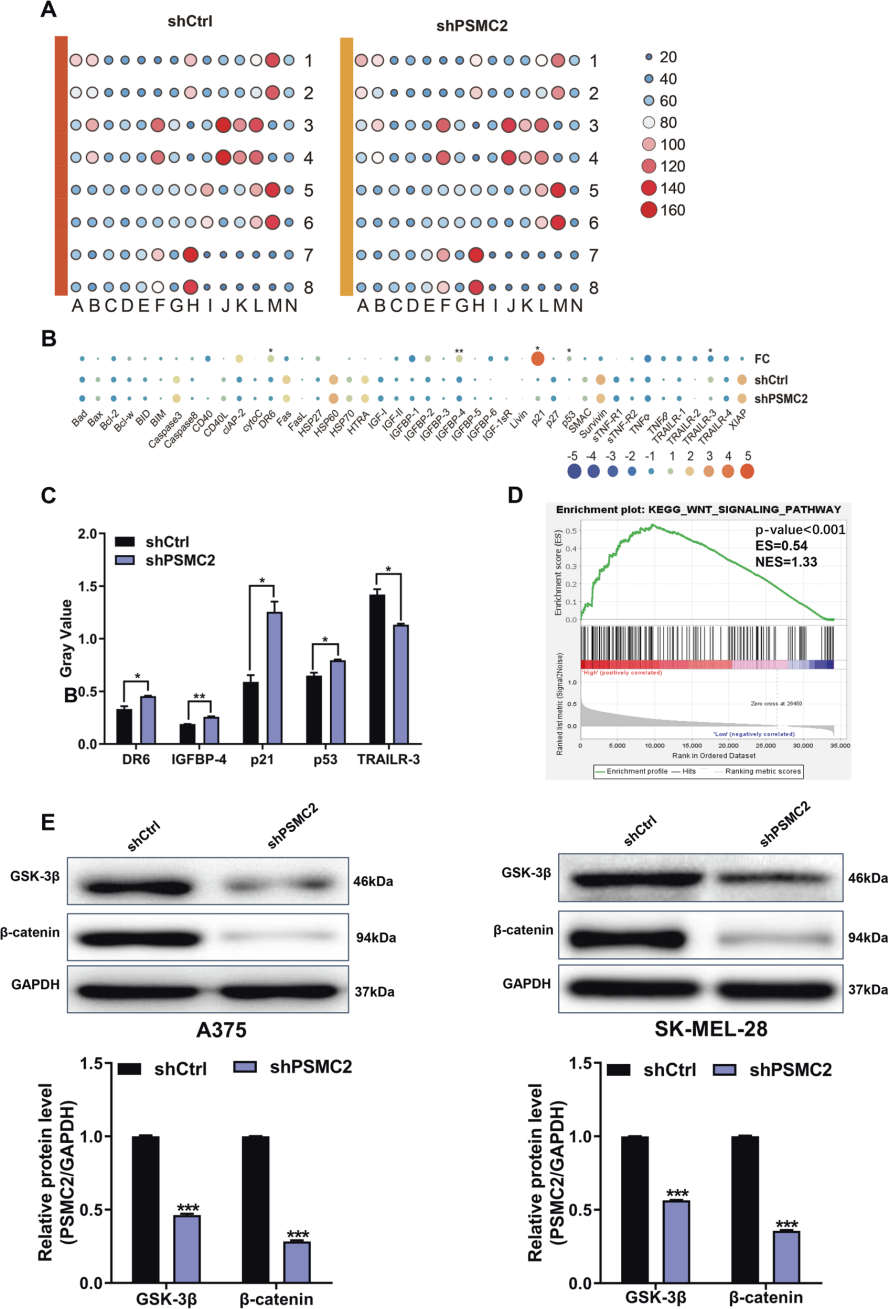
Exploring the downstream regulatory mechanism and signaling pathway of PSMC2 in SKCM cells. **A** Human apoptosis antibody array was performed to analyze the differential expression of human apoptotic markers in SK-MEL-28 cells between shPSMC2 and shCtrl groups. **B** The relative protein levels of 43 human apoptotic markers in SK-MEL-28 cells between shPSMC2 and shCtrl groups. FC, fold change. **C** Gray value analysis for five differential expression proteins (DR6, IGFBP-4, p21, p53, and TRAILR-3) in SK-MEL-28 cells between shPSMC2 and shCtrl groups. **D** Pathway enrichment gene sets of the Wnt signaling pathway in SKCM were shown in snapshoot of GSEA. **E** Western blot was used to detect the expression of GSK-3β and β-catenin in A375 and SK-MEL-28 cells after shPSMC2 transfection. Each independent experiment was repeated at least three times. Data were presented as mean ± SD from three independent experiments. *p < 0.05, **p < 0.01 and ***p < 0.001.

To further elucidate the underlying molecular mechanism of PSMC2 in direct SKCM progression, a protein chip assay was conducted to discover genes affected by PSMC2 silencing. As p21 was one of the most strongly upregulated genes and p53 was upregulated at the same time, we mainly focused on the role of the p53/p21 axis in this study. As shown in Fig. 6A, B, we found that the differential expression of 43 human apoptotic-related proteins in SK-MEL-28 cells after PSMC2 knockdown was analyzed using a human apoptosis antibody array. Moreover, the pro-apoptotic proteins were overtly increased includes DR6, IGFBP-4, p21, and p53, while anti-apoptosis protein TRAILR-3 was obviously decreased in the shPSMC2 group comparing to the shCtrl group (Fig. 6C). Notably, p21 and p53 were most strongly upregulated among these pro-apoptotic proteins. Collectively, these results indicated that PSMC2 was likely involved in apoptosis-related signaling pathways, especially the p53/p21 signaling pathway, in SKCM progression. These results match the previous cellular experiments, especially the cell apoptosis assay.

To confirm the downstream associated molecular mechanisms underlying PSMC2 in SKCM cells, the enrichment analysis of related-pathway was performed using GSEA. The GSEA enrichment snapshot showed that most genes involved in the Wnt signaling pathway were found in the region related to PSMC2 in SKCM cells (Fig. 6D). To further verify the GSEA results, we performed western blotting and discovered the proteins expression levels of GSK-3β and β-catenin, key genes of the Wnt signaling pathway, were all significantly decreased in PSMC2 knockdown cells in comparison with control cells (Fig. 6E). Combining these bioinformatics results, we speculated that the Wnt signaling pathway could influence the functions of PSMC2, thereby participating in SKCM progression.

## Discussion

SKCM is a complex disorder characterized by high heterogeneity that places it among the most aggressive type of cancer [4], however related research about functional verification and mechanism of SKCM2 in human cancers has still been lacking. In the present study, we aimed to verify the function of PSMC2 on the malignant biological behaviors of SKCM. By conducting gene microarray analysis in the TCGA database and using IHC, we evaluated the upregulation of PSMC2 in SKCM tissues and found that high PSMC2 expression was closely correlated with the pathological stages and lymphatic metastasis of SKCM patients. Then, we demonstrated that PSMC2 expression was necessary for SKCM cell malignant progressions by reporting that PSMC2 was over-expressed in SKCM cell lines, which was necessary for cell proliferation, migration, apoptosis, and cell cycle regulation. Further GSEA pathway enrichment and western blot analysis revealed that the function of PMSC2 was closely related to the Wnt signaling pathway in SKCM progression. Taken together, we provided compelling evidence for the role of PSMC2 in tumorigenesis and progression of SKCM via regulating the Wnt signaling pathway, supporting that PSMC2 may be an oncogenic gene participating in SKCM progressions.

High PSMC2 expression was closely correlated with the pathological stages and lymphatic metastasis of SKCM patients. A survival analysis showed that the OS of high expression PSMC2 in colorectal cancer samples was lower than that with low expression PSMC2 [8], and patients with pancreatic cancer who expressed high PSMC2 had a poor prognosis [10]. Similarly, in our study, PSMC2 was closely associated with the pathological stages and lymphatic metastasis (N) of SKCM patients, and that PSMC2 expression was ubiquitously expressed in SKCM samples than in the adjacent para-cancer skin tissues, and overexpressed SKCM cell lines, as A375, SK-MEL-1, and SK-MEL-28 than PIG1 cell line. This suggests that PSMC2 may work as a biomarker protein, and can be used as the basis for early diagnosis of SKCM.

Here we suggest that PSMC2 knockdown remarkably inhibited SKCM cell DNA damage, caused SKCM cells cycle arrest in the G2 phase, and upregulated p53 and p21 expression by three evidence. First, besides inhibited cell proliferation, migration in vitro and inhibited tumor growth in vivo, we found that PSMC2 knockdown remarkably inhibited the DNA damage of SKCM cells. Among the expression decreased DNA damage-related proteins, as ATM, γ-H2AX, and CDKN1A after PSMC2 knockdown in this study, CDKN1A was reported to play a critical role in DNA replication and cell-cycle progression at the G1/S checkpoint [14]. Second, the deficiency of PSMC2 also induced apoptosis and cell cycle arrest in the G2 phase. In our study, cell apoptosis induced by PSMC2 knockdown is complex and involved in multi-apoptotic proteins. He et al. found that silencing of PSMC2 significantly suppressed cell proliferation, enhanced apoptosis, and accelerated G2 phase and/or S phase arrest in CC cells [8]. Dissimilarly, our findings revealed that PSMC2 knockdown leads to apoptosis acceleration and cell cycle arrest only in the G2 phase of SKCM cells. Third, among the expression upregulated human apoptotic-related markers, as DR6, IGFBP-4, p21, p53, and TRAILR-3 after PSMC2 knockdown, p21, a CDK inhibitor, was the most significant upregulated, and was reported to participate in cell cycle progression and could play a crucial role in tumor development by the p53 pathway [15–18]. Also, a previous study held that increased expression of PSMC2 was determined in tumor tissues from the p21-HBx transgenic mice [11]. After DNA damage, many cells appear to enter a sustained arrest in the G2 phase of the cell cycle [19]. Escape from apoptosis is an inducible factor for the occurrence of cancer [12]. Additionally, the G2/M checkpoint and DNA repair mechanisms deal with oxidative stress-induced DNA damage, and G2 arrest induced by oxidative stress suppressed CDK1 activity via p53/p21 pathways in mouse zygotes [20]. A previous study had shown that p21 was partially involved in the proximal tubular cells G2 arrest during the initial stage of the renal damage, thereby safeguarding kidney functions [21]. In glioma, an arrest at the G2/M phase of the cell cycle accompanying by an increased expression of p53 was observed [22]. It had been confirmed that cell cycle arrest following p53 activation might occur at the G1/S or G2/M checkpoints [23]. Therefore, we guess that PSMC2 promotes cell cycle progression by regulating the p21/p53 pathway. These findings reveal a new mechanism of PSMC2 to help develop a new target for SKCM treatment, and a better understanding of the downstream signaling pathway of PSMC2 will also contribute to the therapy for SKCM.

We found that the Wnt signaling pathway was enriched in SKCM when PSMC2 was overexpressed and Wnt-related proteins expression, including GSK-3β and β-catenin, and was suppressed when PSMC2 was depletion. Previous studies indicated that the proteasome activator subunits were significantly enriched in several biological processes and pathways, including regulation of autophagy, the cell cycle, apoptosis, and the Wnt pathways in SKCM [24]. The mutated Wnt/β-catenin pathway components were causative to multiple growth-related pathologies and to several cancers [25]. Overall, PSMC2 might participate in a positive regulation to promote the progression of SKCM through regulating the Wnt signaling pathway.

In summary, knockdown of PSMC2 inhibited proliferation and migration, promoted apoptosis, and caused cell cycle arrest in the G2 phase in human SKCM cells. Interestingly, apoptosis-related downstream of p53/p21 signaling pathways and the Wnt signaling pathway underlying PSMC2 was highlighted in the present study, which provides a novel insight into the mechanism of PSMC2 in SKCM. Notably, these findings have important preclinical implications and provide a potential therapeutic target for the clinical treatment of human SKCM.

## Materials and methods

### TCGA data analysis of PSMC2 expression in SKCM

This study made use of data from public databases. In order to determine the expression level of PSMC2 in SKCM, we performed the biological information analysis using The Cancer Genome Atlas (TCGA) database. The Mann-Whitney U analysis was performed to assess the relationship between PSMC2 and the clinical characteristics of patients with SKCM.

### Patients and tissue samples

One hundred five cases of SKCM tissues and adjacent normal tissues (ANT) were obtained from patients hospitalized in the Zhongshan Hospital, Fudan University, and no patients received radiotherapy or chemotherapy before surgical excision. Two pathologists independently diagnosed the histopathological features of SKCM tissues. This study was approved by the Ethics committee of the Research Department of Zhongshan Hospital Biomedical and conformed to the ethical principles set forth by the Declaration of Helsinki. Written informed consents were signed and obtained from all participants.

### Immunohistochemistry (IHC) staining

SKCM tissues and ANT tissues were frozen and cut into 4–8 μm sections at room temperature for 30 min. IHC staining is performed as described previously [26]. Briefly, sections were incubated with primary antibody against PSMC2 overnight at 4 °C. Subsequently, sections were incubated with a specific secondary antibody for one h at room temperature, followed by incubation with horseradish peroxidase (HRP)-labeled streptavidin for one h, then staining with diaminobenzidine (DAB) for 5 min and counterstaining with hematoxylin (Gene Tech, China). The results of IHC staining were assessed blindly by two experienced pathologists. Five randomly visual fields in each section were selected and then assessed by a light microscope to detect PSMC2 positive expression in SKCM tissues with a color of brownish-yellow regarded as positive. Average positive expression rate= positive cells/total cells %.

### Cell culture

Three human SKCM cell lines (A375, SK-MEL-1, and SK-MEL-28), and human normal epidermal melanocytes (PIG1) were all purchased from the American Type Culture Collection (ATCC). A375 and SK-MEL-1 cells were cultured in Dulbecco’s modified Eagle’s medium (DMEM; Gibco, USA) with 20% FBS. SK-MEL-28 cells were cultured in MEM with 10% FBS. PIG1 cells were grown in the medium 254 (Cascade Biologics, USA). All cells were maintained in an atmosphere with 5% CO_2_ at 37 °C. All cell lines used in this study passed STR identification by the purchase agency.

### Lentiviral shPSMC2 construction

Small hairpin RNAs (shRNAs) was designed to target the human PSMC2 gene (Gene ID, 5701) according to its sequence in the NCBI database as following:

shPSMC2-1, 5’-GAGCTCACTGGTATTAAAGAA-3’;

shPSMC2-2, 5’-CAACGTAAAGCAGTTTGCCAA-3 ‘;

shPSMC2-3, 5 ‘-AAGCAAGTTGAAGATGACATT-3’.

Lentivirus without shRNA insert was used as a control. The fragments of shRNA were inserted into the lentivirus vector and transfected into 293 T cells with packaging vectors using Lipofectamine 3000 (Invitrogen) as soon as cell density reached 70%. The recombinant lentivirus (OE-PSMC2) containing PSMC2 were purchased from GeneChem (Shanghai, China). After lentivirus construction, cells were transfected with lentivirus for establishing the over-expressing PSMC2 SKCM cells. After 48 h of transfection, the recombinant lentivirus was collected from a cell culture medium for further infection. Lentivirus was concentrated using the centrifugal ultrafiltration device (Millipore), and then stored at −80 °C.

### CCK-8 assay

CCK-8 assay was performed to assess the cell proliferation of SKCM cells. Briefly, 48 h after infection, cells were collected and plated into 96-well plates (2 × 10^3^ cells/per well). CCK-8 solution was added to each well (containing 200 µL medium) and further cultured for 2 h at 37 °C. The absorbance at 450 nm was detected under an absorbance microplate reader. This absorbance is directly proportional to the number of living cells in the following five days.

### Wound Healing

After infection for 48 h, a total of 100 µL cells were collected and seeded into 24-well plates with a density of 5 × 10^5^ cells/ml, followed by routine culture to form a cell monolayer. Then, cells were scratched, washed, cultured with the medium containing 10 g/L bovine serum albumin (BSA) and 1% FBS, and placed under a microscope for measuring the migration distance. Cells were then continuing to maintain in the medium containing 10% FBS. After 8–24 h, the relative distance of cells migrating to the wounded area was observed again under a microscope.

### Transwell assay

After being starved for 24 h in the serum-free medium, cells were washed with PBS twice and then resuspended with serum-free Opti-MEM (Invitrogen) with the cell density of 5 × 10^4^ cells/ml. Then, cells were resuspended and seeded into the upper chambers of the 24-well Transwell plate (8 µm, Corning Incorporated, Corning, NY), with triplet for each sample and 100 µL in each well. Next, 600 µL of DMEM or MEM culture medium was added into the lower chamber. After incubation for 48 h, cells were fixed with 4% paraformaldehyde for 10 min, washed with PBS three times, and stained with crystal violet for an additional 10 min. After washing with PBS and dried, cell invasion was observed under a microscope, and five random sights were selected to count the cells that crossed the membrane.

### Apoptosis and cell cycle assay

After lentivirus infection, A375 and SK-MEL-28 cells were assessed with a flow cytometer (BD Biosciences, San Diego, CA, USA) following the manufacturer’s instruction. Briefly, cells were trypsinized, washed with PBS, and centrifugated at 1300 rpm for 5 min. Subsequently, cells were washed once in 1× binding buffer and resuspended in 1× staining buffer. 100 µL cell (5 × 10^5^ cells) were then incubated with 10 µL Annexin V-APC apoptosis detection Kit II or 50 µg/mL PI mixed with 0.1 mg/ml RNaseA and 0.1% Triton X-100 at room temperature for 15 or 30 min in the dark. Cell apoptosis and the percentage of cells in a different phase (G1, S, and G2) were analyzed by Cell Quest software (BD Biosciences, USA).

### Xenotransplant murine models

We carried out animal xenograft model studies by institutional guidelines; The right armpit region of 6-week female BALB/c nude mice was given the subcutaneous injection with 0.2 mL exponentially growing lentivirus infected A375 cells (2 × 10^6^ cell/ml). When the tumors reached an average of 100 mm^3^, the mice were randomly divided into two groups (*n* = 10 mice in each group). On the 10th day after injection, we measured tumor diameters every four days by a caliper. Mice were euthanized on the 35th day after injection. Then the tumors were obtained and weighted following necropsy. Finally, the formula (length × width^2^ × ½ mm3) was used to calculate tumor volume at days 10, 14, 18, 22, 26, 30, and 35 post tumor inoculations. All animal procedures were approved by the Institutional Animal Care and Use Committee of the Research Department of Zhongshan Hospital Biomedical.

### Inhibition of proteasome in SKCM cells

The most common agent to inhibit proteasome in experiments is MG132 (a selective proteasome inhibitor, Sigma-Aldrich). Subsequently, A375 and SK-MEL-28 SKCM cells treated with MG132 (30μm/L) were regarded as proteasome-blocking cells.

### Protein chip assay

After shPSMC2 transfection, the apoptosis signaling pathways underlying PSMC2 in SK-MEL-28 cells were detected by the Human apoptosis antibody array Kit (Abcam, Cambridge, MA) according to the manufacturer’s protocols.

### Gene set enrichment analysis (GSEA)

Gene Set Enrichment Analysis (GSEA) (http://software.broadinstitute.org/gsea) was conducted based on the pathway gene set Kyoto Encyclopedia of Genes and Genomes (KEGG) (https://www.kegg.jp/kegg/), which were used to implement gene set enrichment analysis. The expression data of total normalized mRNAs were uploaded to the GSEA v3.0 software. The default weighted enrichment statistic was adopted to process the data 1000 times, with normalized *P* < 0.05 considered to be significantly enriched. Next, the highest upregulated results of the GSEA reports were selected to undergo graphics processing using the “ggplot2” package in R.

### RNA extraction and RT-qPCR

For total RNA extraction, tissues and cultured cells were directly lysed using TRIzol reagent (Thermo Fisher Scientific) after medium removal. The extracted RNA was reverse-transcribed into cDNA using a PrimeScript™ RT Reagent Kit (TaKaRa), with the manufacturer’s instructions. The obtained cDNA samples were diluted and used for real-time quantitative PCR (RT-qPCR) with SYBR^®^ Premix Ex Taq™ II (TaKaRa) (Thermo Fisher Scientific). GAPDH was measured as the internal control. The relative expression level of genes was calculated using the 2^–△△Ct^ method.

### Protein extraction and Western blot analysis

RIPA lysis buffer supplemented with protease inhibitor (Roche) and phosphatase inhibitor (Roche) was directly added to cell layers and extracted total proteins. Protein concentrations were determined using the BCA protein assay kit (Beyotime). Equal volume and quantity of protein samples were electrophoresed on 10% SDS-PAGE and then transferred onto polyvinylidene difluoride (PVDF) membranes (Millipore). The membranes were blocked with 5% skimmed milk in TBST at room temperature for one h and incubated with appropriate primary antibodies at 4 °C overnight. The next day, the membranes were washed with TBST three times and incubated with appropriate secondary HRP antibodies at room temperature for one h. The membrane was rewashed with TBST three times, followed by incubation with appropriated HRP-conjugated secondary antibody. The subsequent immunoblot analysis was performed using enhanced chemiluminescence (ECL). The protein levels were normalized to GAPDH, and the densitometry was measured using Image J software.

### Statistical analysis

The data are expressed as the mean ± standard deviation (SD) and analyzed by GraphPad Prism 8.0 software. The statistical significance of the data between groups was compared using Student’s *t*-test or one-way ANOVA. Relationship between PSMC2 expression and clinical characteristics in patients with malignant melanoma using Mann–Whitney U analysis. *P* < 0.05 was defined as statistically significant.

## Supplementary information

original data of all protein bands

Author Contribution Statement

## Data Availability

The datasets used and/or analyzed during the current study are available from the corresponding author on reasonable request.
