# Supplementary material for: PSMC2 knockdown suppressed tumor progression of skin cutaneous melanoma
Source: Cell Death Discov. 2021 Oct 29;7:323. doi: 10.1038/s41420-021-00727-2 (PMC8556233; doi:10.1038/s41420-021-00727-2)
Supplement: Supplementary file 2 — Author Contribution Statement [file 41420_2021_727_MOESM2_ESM.pdf]

DECLARATION OF CONTRIBUTIONS TO ARTICLE

ADMC

Manuscript Number:

Journal Name: *Cell Death Discovery* (the 'Journal')

Proposed Title of the Contribution:

PSMC2 knockdown suppressed tumor progression of skin cutaneous melanoma via Wnt signaling pathway (the 'Contribution)

Author(s):

Yanwen Yang, Fazhi Qi, Chuanyuan Wei, Jiaqi Liu, Yong Zhang, Wenjie Luan, Jianying Gu (the 'Authors')

For all *CDDis*covery articles, each person named as an author in the published version must be able to show he or she has contributed substantially to the article.

Authorship credit should be based on 1) substantial contributions to conception and design, acquisition of data, or analysis and interpretation of data; 2) drafting the article or revising it critically for important intellectual content; and 3) final approval of the version to be published. Authors should meet conditions 1, 2 and 3.

Any person who cannot be shown to have made a substantial contribution to the article cannot be listed as an author in the final version. The name of any person who is deemed to have made a minor contribution can, however, appear in the Acknowledgments section of the article.

Please complete the table below to indicate the contributions of all named authors to the manuscript.

Author Full Name:	Specification of Contribution to the Manuscript:
Yanwen Yang	designed this work and wrote the main manuscript text;performed the experiments;prepared figures
Fazhi Qi	designed this work and wrote the main manuscript text
Chuanyuan Wei	performed the experiments.
Jiaqi Liu	performed the experiments.
Yong Zhang	performed the experiments.
Wenjie Luan	prepared figures.
Jianying Gu	prepared figures.

Please complete the table below to indicate the contributions of all named authors to the figures.

Figure 1:

Yanwen Yang, Wenjie Luan and Jianying Gu contributed to prepare the figure.

Figure 2:

Yanwen Yang, Wenjie Luan and Jianying Gu contributed to prepare the figure.

Figure 3:

Yanwen Yang, Wenjie Luan and Jianying Gu contributed to prepare the figure.

Figure 4:

Yanwen Yang, Wenjie Luan and Jianying Gu contributed to prepare the figure.

Figure 5:

Yanwen Yang, Wenjie Luan and Jianying Gu contributed to prepare the figure.

Figure 6:

Yanwen Yang, Wenjie Luan and Jianying Gu contributed to prepare the figure.

Signed for and on behalf of the Author(s):	Print Name:	Date:
Fazhi Qi	Fazhi Qi	2021.4.20
